# Psychometric properties of the contextual body image questionnaire for athletes: a replication and extension study in female collegiate athletes

**DOI:** 10.1186/s40337-021-00414-8

**Published:** 2021-05-05

**Authors:** Tiffany Stewart, Lisa Kilpela, Nicole Wesley, Kate Baule, Carolyn Becker

**Affiliations:** 1 Pennington Biomedical Research Center, Baton Rouge, LA USA; 2 UT Health San Antonio, San Antonio, TX USA; 3 Trinity University, San Antonio, TX USA

**Keywords:** Body image, Athletes, Sports, Eating disorders, Depression

## Abstract

**Background:**

Although the link between body dissatisfaction and eating disorder (ED) pathology is well-established in general female samples, less is known about contextual body image (CBI) among female athletes. CBI refers to female athletes’ body image concerns in two contexts: sport and daily life. The Contextual Body Image Questionnaire for Athletes (CBIQA) measures four dimensions of body image (Appearance, Thin-Fat Self-Evaluation, Thin-Fat Others’ Evaluation, and Muscularity) in both contexts. In a sample of female collegiate athletes, this study sought to A) investigate the psychometric properties of the CBIQA, B) examine the cross-sectional relation of CBI with ED pathology and negative affect, and C) assess the degree to which CBI prospectively predicts ED pathology and negative affect.

**Method:**

Using self-report data collected from a multi-site parent trial, we examined the psychometric properties of the CBIQA by confirmatory factor analysis. We assessed construct and criterion validity via cross-sectional bivariate correlation analyses with thin-ideal internalization, negative affect, and ED pathology. Using data from Time 1 and 6 months later (Time 2), we investigated the degree to which CBI prospectively predicted ED pathology and negative affect.

**Results:**

Results from the CFA largely confirmed de Bruin et al.’s (2011) original factor analysis. Two CBIQA dimensions (Thin-Fat Self and Appearance) in both contexts correlated with ED pathology and negative affect. Thin-Fat Others also correlated with ED pathology in both contexts and negative affect in the sport context. The Muscularity dimension was predominantly orthogonal with other measures. CBIQA dimensions were uncorrelated with thin-ideal internalization. When controlling for BMI and Time 1 scores, daily life and sport appearance concerns predicted ED pathology, whereas perceived evaluation of thin-fat by others in the sport context predicted negative affect 6 months later.

**Conclusions:**

Results support the psychometric validity of the CBIQA and suggest that it captures variance discrete from thin-ideal internalization. The Muscularity dimension largely was not related to other outcomes. Further, specific elements of perceived self- and other-evaluation in both contexts is relevant to risk for ED pathology and negative affect. Future research could examine the impact of dual body image between sports seasons and after transitioning out of sport.

**Clinical trials registration:**

NCT01735994.

## Plain English summary

Female athletes evaluate their bodies in two separate contexts, based on their identities in their sport and daily life: this is called contextual body image (CBI). The Contextual Body Image Questionnaire for Athletes (CBIQA) measures four specific elements of CBI among athletes. This study found that the CBIQA measures its intended four elements of CBI, and that it measures an aspect of body image that is different from traditional thin-ideal internalization. Except for evaluation of Muscularity, the elements of CBI were also related to eating disorder measures. Lastly, taking into account earlier scores on the two outcomes (eating disorder behaviors and negative mood), appearance concerns in both daily life and sport predicted eating disorder symptoms 6 months later. Only perceived evaluation of being fat in sport predicted negative mood 6 months later. Our findings suggest that the CBIQA is measuring body image elements that are unique from other types of body image measures, but are still related to eating disorder symptoms, among female competitive athletes. More research is needed to better understand how athletes are affected by body image concerns in both contexts of their identity.

## Introduction

Contextual body image (CBI) refers to the dual nature of female athlete body image, which consists of body image in sport and in everyday life [1]. Body dissatisfaction has been identified as a significant risk factor for numerous negative outcomes (e.g., depression, eating disorders (ED), unhealthy weight control behaviors) in non-athletic populations [2, 3]; yet the association of CBI with negative outcomes has received less attention. One challenge in studying CBI has been the lack of a well-established measure. Recently developed by de Bruin and colleagues [1], the Contextual Body Image Questionnaire for Athletes (CBIQA) assesses various aspects of body image within these two contexts (i.e., sport and daily life). In the only validation study to date, de Bruin et al. [1] found that the CBIQA assessed four dimensions for each of two body image contexts (i.e., sport and daily life). The sport body image context refers to body evaluation within athletic circumstances whereas the daily life body image context refers to body evaluation in everyday life. The four dimensions in both of these body image contexts assess the following: (1) “Appearance” - Evaluation of appearance (ugly to beautiful); (2) “Muscularity” - Level of muscularity (unmuscular to muscular); (3) “Thin-Fat Self” - Self-evaluation of shape/weight/fat (thin/low to fat/high); and (4) “Thin-Fat Others” - Perceived opinions of others on shape/weight/fat (thin/low to fat/high). For the “Appearance” dimension, participants rate themselves from “very ugly” to “very beautiful”. For “Muscularity”, participants rate the muscularity of their body from “much too unmuscular” to “much too muscular” compared to others. For “Thin-Fat Self”, participants rate their body weight and body fat percentage from “much too low” to “much too high”. Finally, for “Thin-Fat Others”, participants rate whether others thought their body weight and body fat percentage was too low, too high, or neither.

Results from the de Bruin validation study also indicated that all four dimensions in the sport context cross-sectionally correlated with the total score from the Eating Disorder Examination Questionnaire (EDE-Q). For the daily life context, both the Appearance and Thin-Fat Self dimensions correlated with the EDE-Q total score. One limitation of this additional set of analyses, however, was that they were conducted in a relatively small sample (*N* = 52) of elite female athletes (i.e., competing at the national or international level) whose sports tend to emphasize leanness (e.g., aesthetic or distance sports). Thus, it is unclear to what degree these findings would generalize to a larger, more inclusive, sample of female athletes.

The aim of the present study was to further investigate the psychometric properties of the CBIQA and to investigate its prospective relation to negative outcomes using data collected as a part of a larger, multi-site, randomized controlled trial, the Female Athlete Body (FAB) Project (baseline *N* = 481: [NIMH 1 RO1 MH094448–01]). Female collegiate athletes at four different universities in the United States completed the CBIQA at 12 months (*n* = 380) and 18 months (*n* = 347) after randomization into the main FAB trial. The objectives of this study were to investigate (1) the psychometric properties of the CBIQA (2) the relationship of CBIQA contexts and dimensions to eating disorder pathology, thin-ideal internalization, and negative affect, and (3) assess the degree to which CBI prospectively predicts ED pathology and negative affect in female collegiate athletes. Hypotheses included: (1) the psychometric properties of the CBIQA would be replicated in our sample, (2) CBIQA dimensions in both contexts (sport and daily life) would demonstrate construct validity at the factor level (i.e., factor validity) convergent with measures of body image (thin-ideal internalization and weight/shape concerns) and would demonstrate concurrent criterion validity with measures of theorized correlates (ED pathology and negative affect), and (3) CBIQA would prospectively predict ED pathology and negative affect 6 months later, thus demonstrating predictive criterion validity.

## Methods

### Study design

The parent study included a four-university, three-site parallel-group randomized control trial comparing the FAB group to a waitlist control group [4]. The FAB group participated in an intervention delivered over 3 weeks and broken into three, 80-min sessions led by their peers. FAB is an interactive, small-group, 3-session manualized intervention that encourages participants to strive for the athlete-specific healthy ideal (defined as the way one’s body appears when one is appropriately striving to simultaneously maximize physical and mental health, quality of life, and athletic performance) instead of an idealized body appearance. Main components of FAB include a focus on the healthy-ideal versus societal and sport-specific thin-ideals, the Female Athlete Triad, nutrition, sleep, and healthy exercise, goal setting, and body image exercise [4]. The waitlist control group received a Female Athlete Triad brochure at the beginning of the study. Data were collected at baseline, 3 weeks (post-intervention for the FAB group), 6 months, 12 months, and 18 months for both groups. For more detailed information on study design, baseline data, and main outcomes, please see Stewart et al. [4, 5].

### Ethics

The study was monitored and approved by the Institutional Review Board (IRB) of the coordinating center at Pennington Biomedical Research Center (Baton Rouge, LA). Each partner site had IRB approval as well. Participants provided informed consent and those under the age of 18 also provided assent. Each participant earned compensation based on completing a packet of questionnaires that included the measures of focus in this paper ($20) and phone interviews ($30) at five time points for a possible total of $250 earned from this study. These incentives were in line with National Collegiate Athlete Association (NCAA) guidelines and provided motivation to student athletes constricted by time and responsibilities. A data and safety monitoring board provided study oversight.

### Recruitment

Participants included female athletes at each university within the ages of 17–27 years (mean age at baseline for the parent study = 19 years); mean BMI at 12-month follow-up (Time 1 for this study) was 22.70 (SD = 2.91).

Study staff worked with the athletic training staff at the university athletic departments to identify the athletic teams who would participate in the study. Additional team meetings were scheduled outside of the athletes’ regularly scheduled team meetings for the sole purpose of study staff meeting with athletes about participation in the study. Thus, study staff recruited participants in the absence of athletics staff at team meetings to limit potential coercion. Study staff informed athletes that study participation was anonymous and voluntary (see Stewart et al.,4, for details). To further reduce coercion, athletes were informed that coaches and athletics staff would not know which athletes participated or declined in the study.

The majority of participants reported White race (81%); 14.8% reported Black race and 13.1% endorsed Hispanic ethnicity. Study enrollment began in August 2012 and completed in October 2014.

### Procedure

As noted above, data for the current study are a part of the larger, parent study. For the parent study, participation in the FAB intervention program was separate from participation in the study (questionnaires and phone interviews) because the athletic departments wanted all athletes to participate in the program. Thus, all athletes participated in the program unless granted an excused absence by athletics staff, but only those who consented to participate in the research study completed the questionnaires and phone interviews. Athletes were allowed to drop out of the study at any time, consistent with similar trials conducted previously [6]. Follow-up data collection was conducted at set times for each group and participants who could not attend completed measures either individually with study staff or electronically via email. Some data were lost to follow-up and those participants were recorded as missing only for that time point, as some participants returned at later follow-up points.

The CBIQA was introduced at the 12- and 18-month follow-up time points of the parent study. For the purposes of this study, data collected at 12-month follow-up in the parent trial serve as Time 1 data (baseline), while data collected at 18-month follow-up of the parent trial (i.e., 6 months later) comprise Time 2.

### Assessments

In addition to the measures listed below, we collected demographics and calculated body mass index (BMI) from self-reported height and weight (see Table 1 for descriptive statistics).

**Table 1:** 12-month (Time 1) and 18-month (Time 2) descriptive statistics

	Time 1 (*N* = 380) *M* (SD)	Time 2 (*N* = 347) *M* (SD)
CBIQA Daily Life		
Appearance	5.15 (.89)	5.20 (.90)
Muscle	4.05 (.68)	4.04 (.67)
Thin-fat self	4.42 (.68)	4.41 (.71)
Thin-fat other	4.02 (.61)	4.04 (.61)
CBIQA Sport		
Appearance	4.97 (.98)	4.98 (1.01)
Muscle	3.81 (.66)	3.78 (.66)
Thin-fat self	4.40 (.72)	4.39 (.72)
Thin-fat other	4.10 (.55)	4.11 (.61)
EDE-Q		
EDE-Q Res	1.04 (1.19)	1.07 (1.16)
EDE-Q EC	.48 (.78)	.54 (.84)
EDE-Q WC	1.06 (1.16)	1.09 (1.26)
EDE-Q SC	1.32 (1.24)	1.39 (1.31)
EDE-Q Global	.97 (.99)	1.02 (1.02)
IBSS-R	3.39 (.65)	3.37 (.65)
PANAS	1.51 (.57)	1.57 (.64)

*Note: CBIQA* Contextual Body Image Questionnaire for Athletes; *EDE-Q* Eating Disorders Examination – Questionnaire; *EDE-Q Res* Restraint subscale; *EDE-Q EC* Eating concerns subscale; *EDE-Q WC* Weight concerns subscale; *EDE-Q SC* Shape concerns subscale; *EDE-Q Global* EDE-Q global score; *IBSS-R* Ideal Body Stereotype Scale – Revised; *PANAS* Positive and Negative Affect Schedule, negative affect subscale

#### Contextual body image

The Contextual Body Image Questionnaire for Athletes (CBIQA), developed by de Bruin [1], was used to assess the differences in body image in athletes when in sport compared to out of sport. This measure uses a 7-point Likert scale, and has been validated as an appropriate tool to measure both body image contexts in athletes [1]. The CBIQA uses two contexts (daily life and sport), and there are four dimensions within each context (Appearance, Muscularity, Thin-Fat Self, and Thin-Fat Others). Lower scores signify perception as “too ugly” on Appearance and “too unmuscular” on the Muscularity dimension. Higher scores on the Thin-Fat Self and Thin-Fat Others dimensions indicate perceiving one as “too fat.” Questions include statements such as “I think my body shape is …” and “I think the muscularity of my body compared to others is …” , with participants selecting where they are on the scale.

#### Eating disorder (ED) pathology

We used the Eating Disorder Examination Questionnaire (EDE-Q [7];), [8], which evaluates eating attitudes and behaviors over the past 28 days, and higher scores indicate greater pathology. The EDE-Q includes four subscales (restraint, eating concerns, weight concerns, and shape concerns) and a global score. We used the EDE-Q global score to measure overall ED pathology in the predictive models. Past research [9] supports the internal consistency of this measure (α = .92) and test-retest reliability (*r* = .90). Current sample internal consistency was good (α = .92).

#### Thin-ideal internalization

We used the Ideal-body Stereotype Scale-revised (IBSS-R [10];) to assess internalization of the traditional thin-ideal. The IBSS-R has demonstrated good internal validity and test-retest reliability in past research, and predictive validity for onset of bulimic symptomatology [9]. Higher scores indicate greater internalization of the thin-ideal, and internal consistency in the current sample was good (Cronbach’s α = .89).

#### Negative affect

The sadness, guilt, and fear/anxiety subscales of the Positive and Negative Affect Scale-revised (PANAS-X; 11) assessed negative affect; higher scores indicate greater negative affect. The negative affect subscale of the PANAS has demonstrated good internal consistency in past research [11], and internal consistency in this sample was good (Cronbach’s α = .93).

### Statistical analysis

To test the validity of the results from de Bruin et al. [1], a confirmatory factor analysis (CFA) was conducted with Time 1 (12-month data from the FAB study). The CFA was conducted using SAS 9.4 software (SAS Institute Inc., Cary, NC, USA). We assessed overall model fit for each context using the following parameters: 1) model χ^2^ (*p* < .05 indicates good fit); 2) comparative fit index (CFI; CFI ≥ 0.90 indicates good fit); 3) root mean square error of approximation (RMSEA; RMSEA < 0.08 indicates good fit); and 4) standardized root mean square residual (SRMR; SRMR < 0.08 indicates good fit). Factor loadings of .7 or higher were considered acceptable [12].

We examined construct validity by conducting cross-sectional (Time 1 data) bivariate correlation analyses with measures of body image (thin-ideal internalization and the weight and shape concerns subscales of the EDE-Q). We examined concurrent criterion validity with cross-sectional (Time 1 data) bivariate correlation analyses with measures of ED pathology (EDE-Q restraint and eating concerns subscales, global EDE-Q, and negative affect), as ED pathology and negative affect are typically theorized as downstream constructs related to body image disturbances. To adjust for multiple comparisons, we used a cutoff of *p* < .01 as the threshold for significance for the correlation analyses. Finally, to examine the degree to which contextual body image prospectively predicts ED pathology and negative affect 6 months later (i.e., predictive criterion validity), we conducted linear regression models using CBIQA dimensions at Time 1 to predict each outcome at 6 months later (Time 2). For predictive models, we controlled for BMI, which is in line with the de Bruin et al. [1] validation paper, as well as Time 1 scores on each dependent variable. Assumptions for analyses were met. We tested for collinearity among predictor variables using variance inflation factors (VIFs) in each model; VIFs greater than 10 suggest collinearity. We also examined tolerance; tolerance below 0.2 indicates multicollinearity. We did not control for intervention group because we did not see a strong rationale that a 3 × 80-min intervention sessions conducted a year earlier would differentially affect the possible predictive relationship between CBI and outcomes. Analyses were completed using SPSS version 26 (IBM SPSS Statistics for Windows, Version 26.0. Armonk, NY: IBM Corp.)

## Results

The ancillary study comprised data from 380 female collegiate athletes with a mean age of 20 years. Results from the CFAs (Time 1 data) largely confirmed de Bruin’s original factor analysis [1] with slightly different factor loadings (Tables 2-3). Indicators of overall model fit were mixed for the daily life context (χ^2^ (84) = 536.32, *p* < .001; CFI = 0.90; RMSEA = 0.10; SRMR = 0.06) and the sport context (χ^2^ (84) =727.13, *p* < .001; CFI = 0.89; RMSEA = 0.14; SRMR = 0.05). Internal consistency in the current sample for all dimensions in the daily life context was good (see Table 2; Cronbach’s α range = .82–.93), as was internal consistency for all dimensions in the sport context (see Table 3; Cronbach’s α range = .87–.95). All factor loadings were significant at the *p* > .0001 level; factor loadings for items exceeded .7, with the exceptions of body shape as perceived by others and muscularity as perceived by others in the daily life context (Table 2), and body shape as perceived by others in the sport context (Table 3).

**Table 2:** Confirmatory factor analysis with factor loadings for daily life context using 12-month (Time 1) data

Factors	Thin-fat dimension		Appearance	Muscularity
	Self	Other		
CBIQA Daily Life				
Appearance^a^			0.871	
Appearance^b^			0.842	
Appearance^c^			0.743	
Body shape^a^	0.766			
Body shape^b^	0.797			
Body shape^c^		0.525		
Muscularity^a^				0.777
Muscularity^b^				0.840
Muscularity^c^				0.650
Body weight^a^	0.801			
Body weight^b^	0.877			
Body weight^c^		0.752		
Fat percentage^a^	0.827			
Fat percentage^b^	0.861			
Fat percentage^c^		0.803		
Reliability (α)	0.934	0.887	0.883	0.822
Variance proportion	39.44%	27.53%	20.45%	12.51%

*Note:* ^a^Own perception; ^b^Own perception compared to others; ^c^Perceived opinion of others

**Table 3:** Confirmatory factor analysis with factor loadings for sport context using 12-month (Time 1) data

Factors	Thin-fat dimension		Appearance	Muscularity
	Self	Other		
CBIQA Sport				
Appearance^a^			0.916	
Appearance^b^			0.919	
Appearance^c^			0.847	
Body shape^a^	0.879			
Body shape^b^	0.899			
Body shape^c^		0.676		
Muscularity^a^				0.808
Muscularity^b^				0.894
Muscularity^c^				0.773
Body weight^a^	0.842			
Body weight^b^	0.847			
Body weight^c^		0.779		
Fat percentage^a^	0.869			
Fat percentage^b^	0.879			
Fat percentage^c^		0.792		
Reliability (α)	0.952	0.896	0.933	0.876
Variance proportion	40.00%	25.75%	21.00%	14.15%

*Note:* ^a^Own perception; ^b^Own perception compared to others; ^c^Perceived opinion of others

Correlation analyses (Table 4) indicated that Appearance, Thin-Fat Self, and Thin-Fat Other dimensions in both body image contexts (sport and daily life) correlated with EDE-Q weight and shape concerns, suggesting convergent construct validity. Appearance, Thin-Fat Self, and Thin-Fat Other dimensions were correlated with EDE-Q restraint and eating concerns subscales, EDE-Q global score, and negative affect (except Thin-Fat Others in daily life), indicating concurrent criterion validity. Traditional thin-ideal internalization as measured by the IBSS-R largely was orthogonal with all dimensions except Muscularity in sport, which indicated a small negative correlation (i.e., more Muscularity in sport was correlated with lower thin-ideal internalization), suggesting discriminant validity. Only within the sport context did the Muscularity dimension correlate with negative affect; no correlations were significant for Muscularity in the daily life context. Thus, three of the CBIQA dimensions (Appearance, Thin-Fat Self, and Thin-Fat Others) within both body image contexts largely correlated with ED pathology and negative affect, while not with thin-ideal internalization.

**Table 4:** Cross-sectional correlations of CBIQA domains with key body image constructs (*N* = 380)

	Daily Life				Sport			
	Appearance	Muscularity	Thin-fat self	Thin-fat other	Appearance	Muscularity	Thin-fat self	Thin-fat other
EDEQ Res	−.232**	.023	.443**	.226**	−.246**	.027	.391**	.276**
EDEQ EC	−.260**	.059	.401**	.280**	−.294**	.015	.369**	.339**
EDEQ WC	−.375**	.016	.620**	.387**	−.383**	.014	.568**	.428**
EDEQ SC	−.391**	−.021	.594**	.385**	−.407**	−.029	.559**	.422**
EDEQ Global	−.354**	.017	.581**	.358**	−.371**	.006	.533**	.408**
IBSSR	.059	−.082	.053	−.086	−.023	−.143**	.048	−.044
PANAS	−.224**	−.125	.222**	.130	−.250**	−.188**	.226**	.163**

Note: ** = *p* < .01; *CBIQA* Contextual Body Image Questionnaire for Athletes; *EDE-Q* Eating Disorders Examination – Questionnaire; *EDE-Q Res* Restraint subscale; *EDE-Q EC* Eating concerns subscale; *EDE-Q WC* Weight concerns subscale; *EDE-Q SC* Shape concerns subscale; *EDE-Q Global* EDE-Q global score; *IBSS-R* Ideal Body Stereotype Scale – Revised; *PANAS* Positive and Negative Affect Schedule, negative affect subscale

Note: appearance lower scores = “too ugly;” muscularity lower scores = “too unmuscular;” thin/fat both dimensions higher = “too fat”

Regarding prospective prediction of ED pathology 6 months later (Table 5), Time 1 daily life and sport Appearance dimensions predicted EDE-Q global scores at Time 2 when controlling for Time 1 EDE-Q global scores and BMI. The overall model accounted for 57.3% (adjusted R^2^) of the variance (*F*(10, 301) = 41.40, *p* < .001). For negative affect, only Time 1 sport Thin-Fat Others predicted negative affect 6 months later (Time 2) when controlling for Time 1 PANAS scores and BMI (Table 6). The overall model predicting negative affect accounted for 37.4% (adjusted R^2^) of the variance (*F*(10, 304) = 19.19, *p* < .001). Durbin-Watson statistics indicated no concern for autocorrelation in either model. VIFs were less than 10 and all tolerance statistics were > 0.2, suggesting no multicollinearity among predictor variables in either model.

**Table 5:** Summary of multiple linear regression predicting ED pathology at 6-month follow-up (Time 2)

	B	SE B	ß	*t* value	Sig
BMI 12mo	−.019	.016	−.053	−1.168	.244
**EDE-Q 12mo**	**.656**	**.050**	**.646**	**13.187**	**.000**
Daily					
**Appearance**	**.168**	**.074**	**.145**	**2.267**	**.024**
Muscle	−.073	.074	−.050	−.985	.325
Thin-fat self	.052	.126	.035	.411	.682
Thin-fat other	.046	.109	.027	.419	.675
Sport					
**Appearance**	**−.206**	**.066**	**−.200**	**−3.118**	**.002**
Muscle	.097	.077	.064	1.255	.210
Thin-fat self	.023	.111	.016	.208	.836
Thin-fat other	.180	.118	.101	1.526	.128

**Note:** Bold indicates statistical significance at α =0.05; *BMI* Body mass index; *EDE-Q* Eating Disorders Examination-Questionnaire global score; 12mo = data collected at 12-month follow-up in the parent trial, comprising Time 1 in the current study; Durbin-Watson test of autocorrelation = 1.8; VIFs ranged from 1.0–5.1; all tolerance values < 0.2

**Table 6:** Summary of multiple linear regression predicting negative affect at 6-month follow-up (Time 2)

	B	SE B	ß	*t* value	Sig
BMI 12mo	−.001	.012	−.003	−.059	.953
**PANAS 12mo**	**.591**	**.050**	**.572**	**11.719**	.000*
Daily					
Appearance	−.007	.054	−.010	−.128	.898
Muscle	−.066	.054	−.074	−1.214	.226
Thin-fat self	−.031	.088	−.035	−.353	.724
Thin-fat other	−.087	.080	−.084	−1.077	.282
Sport					
Appearance	−.029	.048	−.046	−.596	.552
Muscle	.012	.057	.013	.218	.827
Thin-fat self	.020	.081	.024	.249	.803
**Thin-fat other**	**.176**	**.086**	**.161**	**2.050**	.041*

**Note:** Bold indicates statistical significance at α = 0.05; *BMI* Body mass index; *PANAS* Positive and Negative Affect Schedule negative affect subscale; 12mo = data collected at 12-month follow-up in the parent trial, comprising Time 1 in the current study; Durbin-Watson test of autocorrelation = 2.0; VIFs ranged from 1.0–4.8; all tolerance values < 0.2

## Discussion

Dual body images for female athletes exist for different contexts, both on and off the field. Traditional gender roles and societal perceptions of women’s sports uniquely affect female athletes’ body image. The female-athlete paradox refers to the conflict women experience between the feminine ideal in daily life and the muscular ideal in athletic domains [13, 14]. Further, women who play sports that are more sexually objectified (e.g., volleyball, gymnastics, tennis) may experience greater thin-ideal internalization and body shame [15]. The specific influences of gender, the thin-ideal, and the athletic ideal may be key to understanding how CBI correlates with eating pathology and negative affect.

The aims of the present study were to further investigate both the psychometric properties of the CBIQA and to explore the degree to which constructs assessed by the CBIQA predicted ED pathology and negative affect 6 months later. With regards to the confirmatory factor analysis, results largely replicated those of de Bruin et al. [1] and provide further support for the psychometric validity of the CBIQA. Similar to de Bruin et al. [1], the item inquiring about body shape as perceived by others in the daily life context had the lowest factor loading (Table 2).

de Bruin and colleagues [1] also examined the association of the four dimensions assessed in daily and sport life with global EDE-Q scores. We sought to both replicate and extend these findings by correlating the dimensions in both contexts with both the global score and the EDE-Q subscale scores. With two exceptions, global score findings were replicated. More specifically, both studies found that the Appearance and Thin-Fat Self dimensions in both contexts correlated with EDE-Q global scores and that daily life Muscularity did not. Findings differed with regards to daily life Thin-Fat Others, which correlated in our study but not de Bruin et al., and sport Muscularity, which correlated in de Bruin, but not our study. One explanation for this may be related to the different nature of our samples as de Bruin’s study comprised more elite athletes, as well as a wider age range (age 11–27), than our sample of collegiate athletes (age 18–21). Further, de Bruin specifically recruited high performance women athletes with disordered eating, whereas our sample had very low rates of disordered eating.

With regard to EDE-Q subscale scores, which were not examined in de Bruin, all dimensions except Muscularity correlated with all subscales in both contexts. Within the Appearance dimension, viewing yourself (and perceiving others’ opinions of yourself) as ugly was related to higher levels of ED pathology in both the daily life and sport contexts. Self-evaluation and perceived other-evaluation as fatter were also related to more ED pathology. Overall, CBIQA dimensions except Muscularity demonstrated construct validity with the weight and shape concerns subscales of the EDE-Q, as well as concurrent criterion validity with ED pathology and negative affect.

Contrary to our hypothesis, traditional thin-ideal internalization was orthogonal to all dimensions except Muscularity in sport, suggesting that the CBIQA is largely tapping into a construct distinct from traditional thin-ideal internalization. Higher Muscularity in sport was associated with lower negative affect and less thin-ideal internalization. Muscularity in daily life appears to be tapping a different construct and was not related to ED pathology, thin-ideal internalization, or negative affect. It was also not predictive of negative outcomes at 6-month follow-up.

In daily life, perceiving oneself as too ugly and too fat was related to higher negative affect. In sport, viewing oneself as uglier, too unmuscular, and too fat (self and other) was related to higher negative affect. Interestingly, lower Muscularity and higher ratings of Thin-Fat Others’ opinions correlated with higher negative affect in the sport context but not in daily life. Muscularity and how others perceive your body may play a larger role in sport than in daily life; the pressures associated with how one’s body is perceived may lead to feeling worse about oneself. The “muscular ideal” and its psychological impact has been studied in men [16], but less so in women. Women show a drive for muscularity much like men, but may be more focused on achieving muscle tone than muscle mass [17].

In addition, the sport setting may particularly emphasize muscle tone and the opinions of others (such as teammates, coaches, and parents) about athletes’ bodies [18]. Feeling criticized or stigmatized in sport (Thin-Fat Others’ opinions) due to body shape and size could affect one’s perception of oneself in the sport overall, which may account for greater negative affect [19]. Despite the association between higher Muscularity and more negative affect, our findings also revealed a small negative correlation between Muscularity in sport and thin-ideal internalization. Athletes who view themselves with higher Muscularity may have less of a drive toward the traditional thin-ideal. In summary, muscularity may be a unique dimension for female athletes that warrants closer examination.

CBIQA scores related to Appearance may predict future ED pathology and negative affect, indicating predictive criterion validity. Therefore, this measure may be helpful in screening for future issues with disordered eating and depression. An athlete-specific screening tool could be useful for preventing and addressing EDs in this population [20]. Further research on contextual body image is needed to evaluate the impact of these dual body images with female athletes-not only while in sport, but while away or retiring from sport, including periods of injury, between seasons and when completing sport.

Strengths of this study include strong internal consistency for all assessments, a large sample, a follow-up time point 6 months later, and the inclusion of prospective analysis to inform about criterion validity. Limitations of the study include reliance on self-report data, attrition at follow-up, and that the CBIQA was delivered to participants 12 months into the FAB program as opposed to the beginning of the parent trial.

## Conclusions

A dual body image exists for female collegiate athletes. In daily life and in sport, different dimensions of body image (Appearance, Muscularity, Thin-Fat Self, and Thin-Fat Others) correlate with ED pathology and negative affect. In both contexts, all dimensions of body image except for Muscularity correlate with more ED pathology. In the sport context, Muscularity and Thin-Fat Others’ opinions may play a more salient role. Appearance-related scores could be used in a screening tool for athletes to predict future disordered eating and depression. This study provides insight into the specific nature of female athlete body image in order to suggest new preventative measures (in terms of EDs and depression) for this population.

## Data Availability

The datasets used and/or analyzed during the current study are available from the corresponding author on reasonable request.

## Abbreviations

BMI: Body mass index
CBI: Contextual body image
CBIQA: Contextual body image questionnaire for athletes
ED: Eating disorder
EDE-Q: Eating disorder examination questionnaire
FAB: Female athlete body project
IBSS-R: Ideal-body stereotype scale-revised
IRB: Institutional review board
NCAA: National collegiate athlete association
NIMH: National institute of mental health
PANAS-X: Positive and negative affect scale-revised
